# Associations between achievement motivation and subjective well-being among medical students: the chain mediating roles of general self-efficacy and psychological resilience

**DOI:** 10.3389/fpsyg.2026.1931480

**Published:** 2026-10-09

**Authors:** Hang Sun, Zhaojin Li, Chuan Gao, Hao Sun, You Zhou, Lanqin Liu, Yanzi Sun, Wenwen Li

**Affiliations:** 1 School of Sports Science, Qufu Normal University, Qufu, China; 2 School of Music, Qufu Normal University, Qufu, China; 3 School of Medical Imaging and Laboratory Science, Jining Medical University, Jining, China

**Keywords:** achievement motivation, chain mediation, general self-efficacy, medical students, psychological resilience, subjective well-being

## Abstract

**Introduction:**

Medical students face prolonged training, rigorous assessment, and professional demands that may affect their subjective well-being. This study aimed to examine the associations between the two dimensions of achievement motivation and subjective well-being among medical students and to investigate the mediating roles of general self-efficacy and psychological resilience.

**Methods:**

A cross-sectional questionnaire survey was conducted among 2,422 medical students in Jining, Shandong Province, China. Structural equation modeling was used to estimate direct and specific indirect associations.

**Results:**

Success motivation had a weak positive direct association with subjective well-being (β = 0.060), whereas failure-avoidance motivation had a weak negative direct association (β = −0.063). Three specific indirect pathways were supported: through self-efficacy alone, resilience alone, and self-efficacy followed by resilience. The total indirect associations were 0.293 and -0.056, accounting for 83.08% and 47.25% of the respective model-based total associations. Among the variables included in the model, resilience had the largest direct association with subjective well-being (β = 0.494).

**Discussion:**

Achievement motivation was associated with medical students’ subjective well-being through efficacy beliefs and psychological resilience. These findings highlight the importance of fostering success-oriented motivation, strengthening general self-efficacy, and cultivating psychological resilience in medical education.

## 1 Introduction

Medical education is generally a lengthy process. Medical students are exposed to a longer training cycle, rigorous program assessments, and stronger professional responsibility and ethical requirements (Dyrbye et al., 2005; Lyndon et al., 2014; Bergmann et al., 2019). Existing studies and systematic reviews indicate that high academic workload and frequent assessments in medical education are associated with relatively high levels of psychological distress among medical students, including depression, anxiety, and burnout (Dyrbye et al., 2006; Rotenstein et al., 2016; Frajerman et al., 2019; Quek et al., 2019). Mental health does not simply refer to the absence of abnormal states; it also includes positive functioning and favorable psychological experiences in life (Keyes, 2002). In positive psychology, subjective well-being is one important construct for understanding this positive psychological state, reflecting individuals’ life satisfaction and positive emotional experiences (Diener, 1984; Diener et al., 1999). Therefore, examining the psychological factors associated with medical students’ subjective well-being has important theoretical and practical significance.

When facing similar academic requirements, medical students may exhibit markedly different emotional experiences and cognitive responses. From the perspective of cognitive appraisal, the same academic demand may be interpreted either as a challenge for self-improvement or as a threat involving external evaluation, setbacks, and failure (Lazarus and Folkman, 1984; Malkoc et al., 2023). This difference may be closely related to achievement motivation. The concept of achievement motivation can be traced to Murray’s early discussion of the need for achievement and was further developed in the work of McClelland et al. (1953), Atkinson (1957), and Murray and McAdams (2007). Based on achievement motivation theory and the approach-avoidance framework, achievement motivation can be divided into the motive to achieve success and the motive to avoid failure (Atkinson, 1957; Elliot and Church, 1997; Elliot and McGregor, 2001). The motive to achieve success reflects the desire to pursue success, cope with challenges, and improve ability, whereas the motive to avoid failure reflects concerns about failure, negative evaluation, and shame. Recent research involving university students suggests that these two motivational tendencies may be associated with subjective well-being in different directions, although the generalizability of this pattern to medical students still requires further verification. According to conservation of resources theory, resource loss is usually more psychologically salient than resource gain (Hobfoll, 1989; Hobfoll et al., 2018). For medical students, the emotional cost of anticipated failure may be more direct and detrimental than the positive benefits associated with pursuing success. Therefore, it is necessary to distinguish the motive to achieve success from the motive to avoid failure when examining the relationship between achievement motivation and subjective well-being.

Self-efficacy may be an important cognitive correlate of achievement motivation and subjective well-being. Social cognitive theory proposes that personal factors influence individuals’ behavior and the environment in which they are situated (Bandura, 1986, 1997). Self-efficacy refers to individuals’ beliefs about whether they can organize and execute the actions required to attain a given level of performance; it focuses on perceived capability rather than overall self-worth (Bandura, 1977; Artino, 2012). Bandura also distinguished self-efficacy from outcome expectations: the former concerns whether one can perform the required action, whereas the latter concerns what is likely to happen after the action is performed. Students with high self-efficacy are more likely to believe that they can complete tasks and master knowledge, whereas students with low self-efficacy are more likely to avoid tasks, reduce effort, or interpret difficulties and emotional arousal as signs of insufficient capability (Bandura, 1997; Artino, 2012). In adolescent and university student samples, self-efficacy is usually positively associated with subjective well-being or life satisfaction (Tong and Song, 2004; Santos et al., 2014; Cattelino et al., 2023). In the context of medical education, success motivation may be associated with stronger efficacy beliefs, whereas failure-avoidance motivation may be associated with lower efficacy beliefs and lower subjective well-being.

Psychological resilience may also be involved in the proposed statistical indirect associations. Psychological resilience refers to individuals’ ability to adapt, recover, and maintain a positive psychological state when they face stress, adversity, or setbacks (Connor and Davidson, 2003). For medical students, resilience is particularly important because the demands of medical training are often continuous rather than temporary (Dyrbye et al., 2005; Yu and Chae, 2020; Duarte et al., 2022). Heavy course burdens, frequent assessments, uncertainty in clinical settings, and professional responsibilities may gradually deplete students’ psychological resources (Dyrbye et al., 2005; Lyndon et al., 2014; Bergmann et al., 2019). From the perspective of conservation of resources theory, resilience can be regarded as a personal resource that helps students maintain and restore mental health functioning under continuous stress (Hobfoll, 1989; Hobfoll et al., 2018; Yu and Chae, 2020). Previous studies have found that psychological resilience is positively associated with subjective well-being or psychological well-being in student and medical-student samples (Neufeld and Malin, 2019; Yu and Chae, 2020; Duarte et al., 2022). Thus, medical students with a stronger motive to achieve success may be more likely to mobilize psychological resilience when facing academic or clinical stress, whereas those with a stronger motive to avoid failure may experience greater resource depletion and weaker recovery capacity.

General self-efficacy and psychological resilience may also be linked within a theoretically specified sequential indirect pathway. Existing studies have shown that general self-efficacy is closely associated with psychological resilience (Sagone and Caroli, 2013; Schwarzer and Warner, 2013; Sagone et al., 2020; Qin et al., 2023). Individuals with low self-efficacy are more likely to develop maladaptive cognitive appraisals and doubt their ability to meet demanding requirements, which may lead to helpless withdrawal rather than active coping (Bandura, 1997; Artino, 2012). In contrast, stronger efficacy beliefs may provide the cognitive foundation for mobilizing resilience and recovering from academic setbacks (Schwarzer and Warner, 2013; Yu and Chae, 2020; Qin et al., 2023). Based on these partial paths, this study further proposed and tested a chain mediation hypothesis: achievement motivation may be associated with subjective well-being through general self-efficacy and psychological resilience. Although previous studies have separately examined the roles of self-efficacy and psychological resilience, few studies have examined this sequential indirect pathway between two-dimensional achievement motivation and subjective well-being among medical students.

Accordingly, based on social cognitive theory and conservation of resources theory, this study specified a cross-sectional chain mediation model to examine the associations between the motive to achieve success and the motive to avoid failure and medical students’ subjective well-being, including statistical indirect paths through general self-efficacy and psychological resilience. The following hypotheses were evaluated within this model (Figure 1):

**Figure 1:**
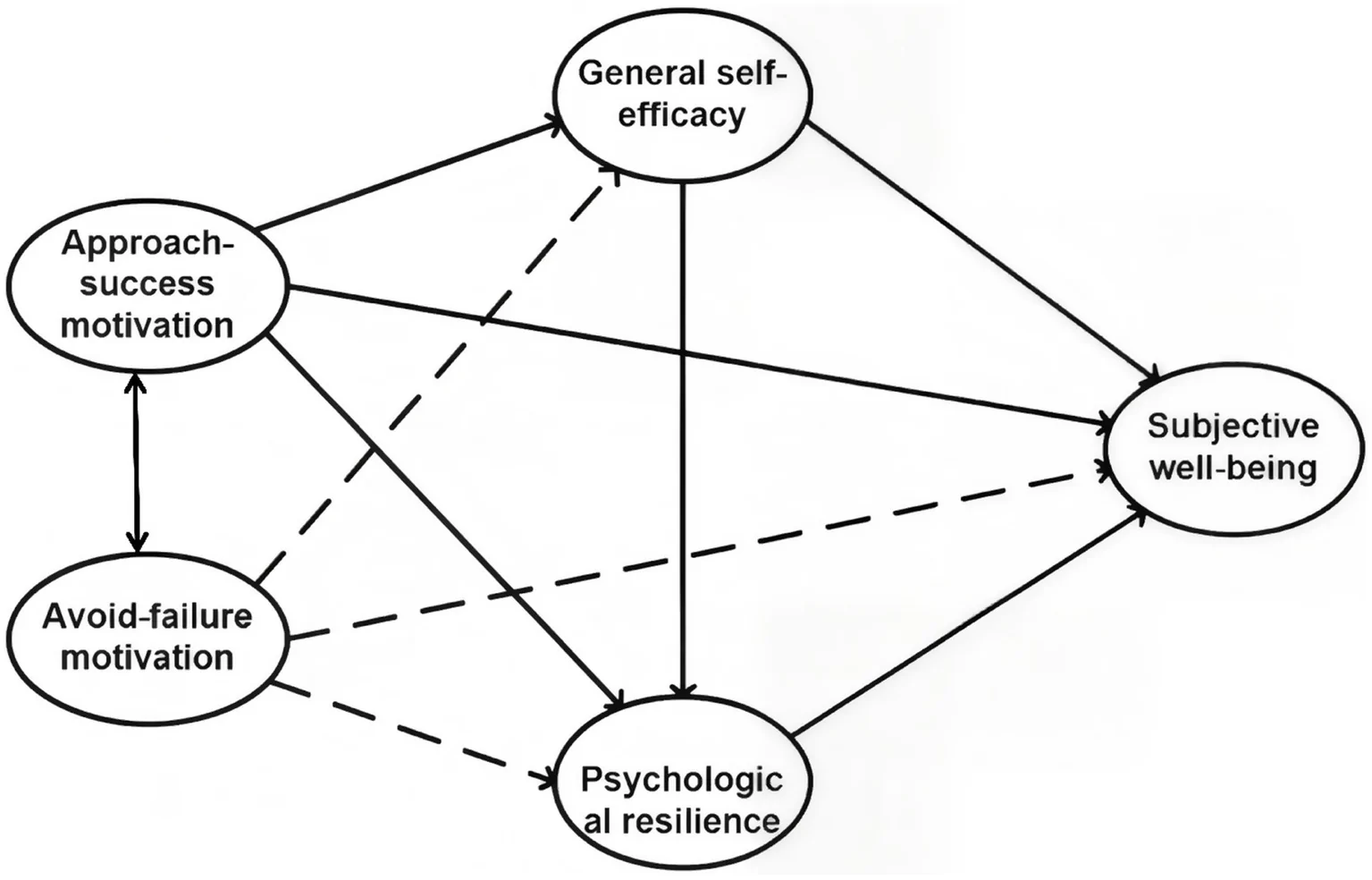
Hypothesized model. Ovals represent latent variables. Solid and dashed single-headed arrows indicate hypothesized positive and negative associations, respectively. The double-headed arrow represents the covariance between the two motivation dimensions.

*H1:* The motive to achieve success is positively correlated with medical students’ subjective well-being, whereas the motive to avoid failure is negatively correlated with subjective well-being.

*H2:* The two dimensions of achievement motivation have statistical indirect associations with subjective well-being through general self-efficacy.

*H3:* The two dimensions of achievement motivation have statistical indirect associations with subjective well-being through psychological resilience.

*H4:* The two dimensions of achievement motivation have statistical indirect associations with subjective well-being through general self-efficacy followed by psychological resilience in the specified model.

## 2 Participants and methods

### 2.1 Research design and participants

This cross-sectional study used convenience sampling to recruit medical students from a medical college in Jining, Shandong Province, China, in 2024. Recruitment was organized by class according to student availability, with greater coverage of the 2024 entry cohort. An approximate recruitment target of 3,000 students was set before data collection. Eligible students completed the questionnaire independently and provided written informed consent.

A total of 3,173 paper questionnaires were returned; 751 were excluded during screening, leaving 2,422 for analysis (76.33% of returned questionnaires). Exclusion reasons are detailed in Section 2.2. Participants were aged 17–21 years (M = 18.93, SD = 1.10). Participant characteristics are presented in Table 1. Only-child status indicates whether a participant had siblings.

**Table 1:** Participant characteristics.

Characteristic	*n* or M (SD)	% or range
Age (years), M (SD)	18.93 (1.10)	17–21 (range)
Male	1,059	43.72
Female	1,363	56.28
Only child	687	28.36
Has siblings	1,735	71.64
No left-behind experience	2,036	84.06
Left-behind experience	386	15.94
Entry cohort: 2022	584	24.11
Entry cohort: 2023	428	17.67
Entry cohort: 2024	1,410	58.22

Percentages use *N* = 2,422. Age is presented as mean (standard deviation) and observed range. Cohort labels denote year of entry.

### 2.2 Survey procedure

The survey was conducted in classrooms. Before questionnaire administration, researchers explained the study purpose and the voluntary nature of participation, including the right to withdraw. All participants provided written informed consent, and no participant withdrew during questionnaire administration. Trained researchers distributed paper questionnaires with the help of student counselors. After collection, the questionnaire responses were entered manually into an electronic dataset.

Screening excluded 315 questionnaires with incomplete scale responses, 30 entirely blank questionnaires, 80 with identical responses throughout an individual scale, 14 with identical responses across all 70 items, and 312 with recorded values outside the permitted item-response ranges. These mutually exclusive categories accounted for all 751 exclusions, leaving 2,422 questionnaires for analysis.

### 2.3 Research instruments

#### 2.3.1 Achievement motivation

Achievement motivation was measured using the 30-item Chinese version of the Achievement Motivation Scale (Nygård and Gjesme, 1973; Ye and Hagtvet, 1992). Items are scored from 1 to 4. Items 1–15 assess the motive to achieve success (MS), and items 16–30 assess the motive to avoid failure (MAF); each subscale sum ranges from 15 to 60. Higher scores indicate a stronger corresponding motivational tendency. The two dimensions were analyzed separately, consistent with the study hypotheses. Cronbach’s α was 0.935 for success motivation and 0.939 for failure avoidance.

#### 2.3.2 Subjective well-being

Subjective well-being was measured using the 20-item Short-Form Subjective Well-Being Scale for Chinese Citizens (SWBS-CC20), developed by Xing (2003). Items are rated from 1 (strongly disagree) to 6 (strongly agree). Under the scoring instructions supplied for this study, items 4, 5, 6, 9, 10, 11, 13, 15, 17, 18, and 20 are reverse-keyed using 7 minus the original response. Higher scores after correct keying indicate greater subjective well-being. The present study focused on overall subjective well-being. Cronbach’s α calculated from the recorded item values was 0.951.

#### 2.3.3 General self-efficacy

General self-efficacy was measured using the 10-item Chinese version of the General Self-Efficacy Scale (Schwarzer and Jerusalem, 1995). Its reliability and validity in Chinese samples have been evaluated by Wang et al. (2001). The scale is unidimensional, and all items are positively keyed. Responses are scored from 1 (not at all true) to 4 (exactly true), yielding a total score of 10–40. Higher scores indicate stronger general self-efficacy. Cronbach’s α was 0.905.

#### 2.3.4 Psychological resilience

Psychological resilience was measured using the 10-item Connor-Davidson Resilience Scale (CD-RISC-10). The original scale was developed by Connor and Davidson (2003), and the 10-item version was developed by Campbell-Sills and Stein (2007). The Chinese 10-item version has been evaluated in nursing students (Ye et al., 2016). Items are scored from 0 (never) to 4 (always), with intermediate responses of rarely (1), sometimes (2), and often (3). The total score ranges from 0 to 40, with higher scores indicating greater psychological resilience. Cronbach’s α was 0.912.

### 2.4 Statistical analysis

Statistical analyses were performed in Python using semopy 2.3.11, and the primary structural model was also reproduced in IBM SPSS Amos. The analysis included 2,422 participants with complete data for all 70 items and 5 composite scores. Descriptive statistics included frequency, percentage, mean, standard deviation, observed range, skewness, and excess kurtosis. Cronbach’s α was calculated for each scale. Pearson correlations were calculated between composite mean scores, with 95% confidence intervals obtained using Fisher’s *z* transformation.

A five-factor confirmatory factor analysis (CFA) included 15 items for the motive to achieve success (AS), 15 for the motive to avoid failure (AF), 20 for subjective well-being (SWB), 10 for general self-efficacy (GSE), and 10 for psychological resilience (PR). Each item loaded on its specified factor, with the first loading fixed to 1. Factor covariances and item residual variances were freely estimated, and cross-loadings and residual covariances were fixed to zero. All 70 items were retained, and no model modifications were applied. Composite reliability (CR), average variance extracted (AVE), and the Fornell–Larcker criterion were evaluated (Fornell and Larcker, 1981; Hair et al., 2019).

Ordinal items were treated as continuous using normal-theory maximum likelihood with a Wishart likelihood objective (MLW). Fit was summarized by the chi-square statistic (χ^2^), degrees of freedom (df), the comparative fit index (CFI), Tucker–Lewis index (TLI), root mean square error of approximation (RMSEA) with a 90% confidence interval, and standardized root mean square residual (SRMR). SRMR was computed separately from standardized covariance residuals; the full specification and calculation conventions are provided in Supplementary Tables S3, S4.

The structural model specified AS and AF as correlated exogenous factors, GSE as regressed on AS and AF, PR as regressed on AS, AF, and GSE, and SWB as regressed on all four other factors. Endogenous disturbances were uncorrelated. An alternative model reversed the order of GSE and PR while retaining the corresponding direct paths and the AS–AF covariance (Supplementary Table S4).

Standardized direct and indirect associations were summarized using 5,000 participant-level nonparametric bootstrap resamples. All item loadings and factor covariances were re-estimated in each resample. Bias-corrected 95% confidence intervals were calculated for the path products and their sums; intervals excluding zero indicated statistical evidence of an association. Each indirect percentage was calculated by dividing the specific or total indirect estimate by the same predictor’s model-based total association and multiplying by 100, conditional on the other motivational dimension.

Additional analyses compared the five-factor measurement model with a one-factor CFA and a four-factor CFA combining GSE and PR. Composite-score regression models were fitted with and without adjustment for age, sex, and entry cohort. Their estimates were compared using 5,000 paired participant-level bootstrap resamples and percentile 95% confidence intervals (Supplementary Table S5). Participants were treated as the independent units of analysis and resampling.

## 3 Results

### 3.1 Preliminary analysis

The five composite scores showed modest skewness and excess kurtosis, with no missing observations in the analysis sample (Table 2; Supplementary Table S1). Cronbach’s α ranged from 0.905 to 0.951.

**Table 2:** Descriptive statistics and Pearson correlations.

Variable	M	SD	1	2	3	4	5
1. AS	2.473	0.587	1.000	–	–	–	–
2. AF	2.117	0.571	−0.138	1.000	–	–	–
3. GSE	2.531	0.580	0.480	−0.155	1.000	–	–
4. PR	2.767	0.590	0.399	−0.134	0.435	1.000	–
5. SWB	2.961	0.550	0.350	−0.161	0.414	0.568	1.000

AS, motive to achieve success; AF, motive to avoid failure; GSE, general self-efficacy; PR, psychological resilience; SWB, subjective well-being. M, mean; SD, standard deviation. Off-diagonal entries are Pearson correlations between composite mean scores; all *p* < 0.001. “–” denotes an omitted duplicate entry. Reliability and validity results are presented in Supplementary Table S2.

Success motivation was positively correlated with subjective well-being (*r* = 0.350, 95% CI [0.314, 0.384]), whereas failure-avoidance motivation was negatively correlated with well-being (*r* = −0.161, 95% CI [−0.200, −0.122]). The association between success motivation and subjective well-being was moderate, whereas the association between failure-avoidance motivation and well-being was weak. Self-efficacy and resilience were positively correlated with well-being; the resilience–well-being correlation was the largest of these correlations (*r* = 0.568). All pairwise correlation tests had *p* < 0.001 (Table 2).

The five-factor CFA had χ^2^(2335) = 2596.375, *p* < 0.001, CFI = 0.997, TLI = 0.997, RMSEA = 0.0068 (90% CI [0.0050, 0.0083]), and SRMR = 0.0167. Standardized loadings ranged from 0.672 to 0.759, CR from 0.905 to 0.951, and AVE from 0.488 to 0.509. The square root of each factor’s AVE exceeded its absolute correlations with other latent factors. Reliability and validity results and item-level estimates are presented in Supplementary Tables S2, S3.

### 3.2 Direct associations in the structural model

The structural model had χ^2^(2335) = 2596.375, *p* < 0.001, χ^2^/df = 1.112, CFI = 0.997, TLI = 0.997, RMSEA = 0.0068 (90% CI [0.0050, 0.0083]), and SRMR = 0.0167. All 5,000 bootstrap resamples converged without inadmissible solutions. The model *R*^2^ values were 0.283 for GSE, 0.278 for PR, and 0.410 for SWB.

The direct associations between success motivation and failure-avoidance motivation and well-being were statistically distinguishable from zero but weak (*β* = 0.060 and −0.063, respectively; Table 3). Figure 2 shows the model’s standardized path coefficients.

**Table 3:** Standardized direct paths in the latent-variable model.

Path	β	Bootstrap SE	95% BC CI
AS → GSE	0.510	0.017	[0.476, 0.543]
AF → GSE	−0.094	0.020	[−0.132, −0.056]
AS → PR	0.244	0.022	[0.196, 0.285]
AF → PR	−0.049	0.019	[−0.087, −0.011]
GSE → PR	0.345	0.023	[0.301, 0.389]
AS → SWB	0.060	0.022	[0.017, 0.103]
AF → SWB	−0.063	0.018	[−0.097, −0.028]
GSE → SWB	0.169	0.023	[0.122, 0.214]
PR → SWB	0.494	0.020	[0.455, 0.533]

AS, motive to achieve success; AF, motive to avoid failure; GSE, general self-efficacy; PR, psychological resilience; SWB, subjective well-being. *N* = 2,422; BC, bias-corrected; CI, confidence interval; SE, standard error. Estimates and SEs are standardized. Intervals were calculated from 5,000 participant-level bootstrap resamples with the measurement model re-estimated. The AS–AF standardized covariance was −0.147.

**Figure 2:**
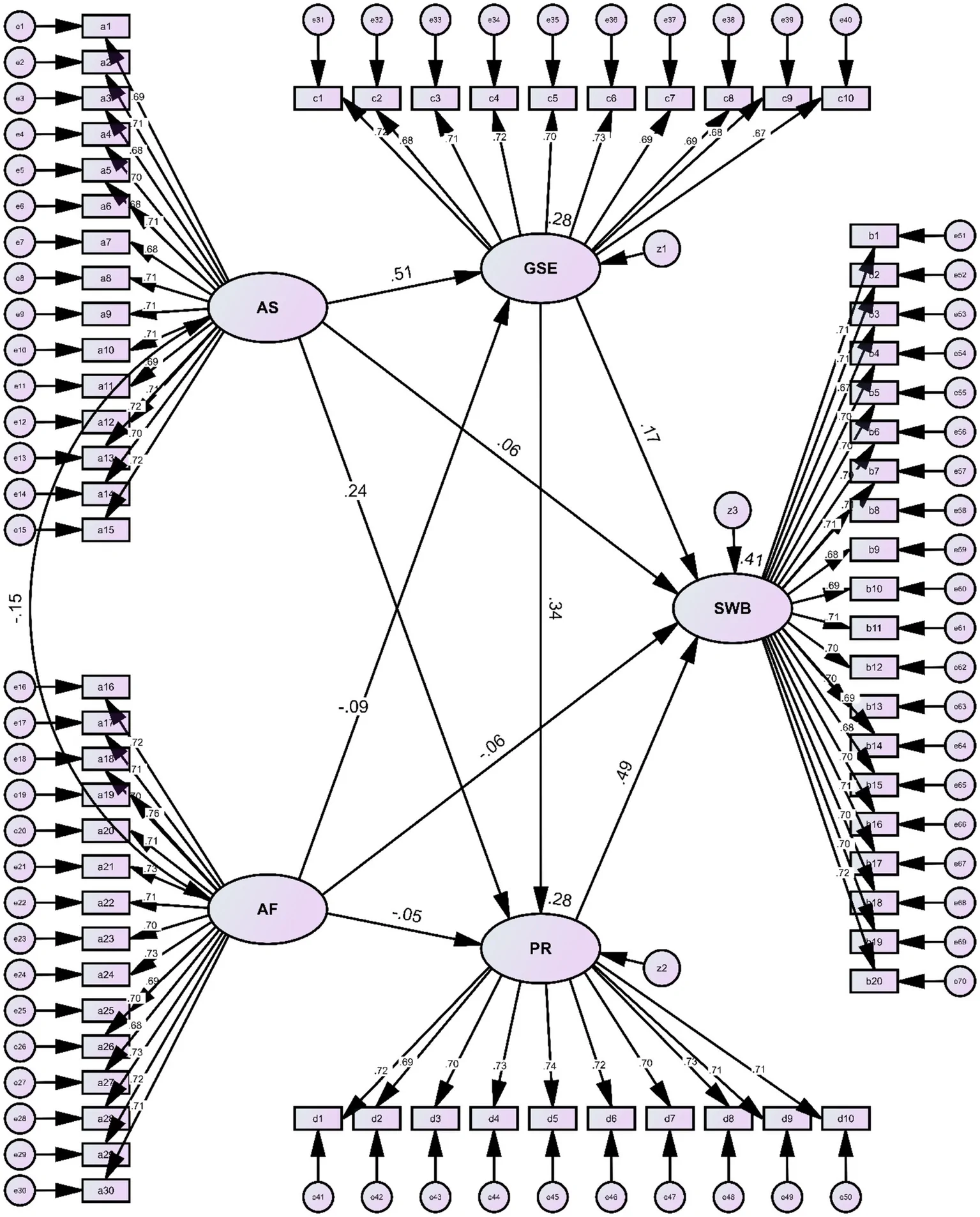
Structural equation model results. Ovals represent latent variables, rectangles represent observed items, and small circles represent residuals. Values on single-headed arrows are standardized factor loadings or path coefficients; the double-headed arrow shows the correlation between AS and AF. Values above GSE, PR, and SWB indicate explained variance (*R*^2^). AS, motive to achieve success; AF, motive to avoid failure; GSE, general self-efficacy; PR, psychological resilience; SWB, subjective well-being.

Among the other paths, AS–GSE (*β* = 0.510) and PR–SWB (*β* = 0.494) were relatively strong within this model; GSE–PR (*β* = 0.345) was moderate. AS–PR (*β* = 0.244) and GSE–SWB (*β* = 0.169) were smaller, while the AF–GSE (*β* = −0.094) and AF–PR (*β* = −0.049) associations were weak.

### 3.3 Statistical indirect associations

Table 4 decomposes each motivational dimension’s model-based total association with well-being into its direct component and three specific indirect components. All six specific indirect estimates had bias-corrected 95% confidence intervals excluding zero. These findings were consistent with the modeled indirect associations proposed in H2–H4.

**Table 4:** Decomposition of model-based associations with subjective well-being.

Component	*β*	Bootstrap SE	95% BC CI	% of total
AS — Via GSE alone	0.086	0.012	[0.062, 0.110]	24.45
AS — Via PR alone	0.120	0.012	[0.096, 0.145]	34.07
AS — Via GSE → PR	0.087	0.007	[0.073, 0.102]	24.56
AS — Total indirect	0.293	0.015	[0.264, 0.322]	83.08
AS — Direct	0.060	0.022	[0.017, 0.103]	16.92
AS — Total	0.353	0.020	[0.312, 0.391]	100.00
AF — Via GSE alone	−0.016	0.004	[−0.025, −0.009]	13.34
AF — Via PR alone	−0.024	0.010	[−0.043, −0.006]	20.50
AF — Via GSE → PR	−0.016	0.004	[−0.024, −0.009]	13.40
AF — Total indirect	−0.056	0.012	[−0.079, −0.033]	47.25
AF — Direct	−0.063	0.018	[−0.097, −0.028]	52.75
AF — Total	−0.119	0.020	[−0.157, −0.077]	100.00

AS, motive to achieve success; AF, motive to avoid failure; GSE, general self-efficacy; PR, psychological resilience; SWB, subjective well-being. *N* = 2,422. Percent of total = 100 × component estimate/total association for the same predictor, conditional on the other motivational dimension. Total indirect is the sum of the three specific indirect estimates; total equals direct plus total indirect. Percentages were calculated before rounding. BC 95% CIs were obtained from the latent-model participant bootstrap.

For success motivation, the paths through GSE alone, PR alone, and GSE followed by PR accounted for 24.45, 34.07, and 24.56% of its total association, respectively. Their combined share was 83.08%, leaving 16.92% as the direct component.

For failure-avoidance motivation, the corresponding shares were 13.34, 20.50, and 13.40%. Their combined share was 47.25%, leaving 52.75% as the direct component.

For both motivational dimensions, the indirect point estimate through PR alone was larger in absolute value than the estimate through GSE alone. Confidence intervals for the component percentages are provided in Supplementary Table S6.

### 3.4 Alternative models and sensitivity analyses

The one-factor CFA (CFI = 0.477, RMSEA = 0.0895) and the four-factor CFA combining GSE and PR (CFI = 0.920, RMSEA = 0.0351) had lower CFI and higher RMSEA values than the five-factor model. Reversing the GSE–PR order yielded the same implied covariance, degrees of freedom, and fit as the primary structural model (Supplementary Table S4). In the composite-score analysis, adjustments for age, sex, and entry cohort changed every standardized path by less than 0.002; the directions and confidence-interval zero-exclusion status of all nine paths and six specific indirect associations were unchanged (Supplementary Table S5).

## 4 Discussion

This study identified contrasting associations between the two dimensions of achievement motivation and subjective well-being and statistical indirect pathways involving general self-efficacy and psychological resilience. These findings connect motivational tendencies with cognitive beliefs and psychological resources in the context of medical education.

### 4.1 The contrasting associations between achievement motivation and well-being

The results showed that the motive to achieve success was positively associated with subjective well-being, while the motive to avoid failure was negatively associated with subjective well-being. This pattern was consistent with achievement motivation theory and with recent evidence from university students showing that the two motivational dimensions may be related to subjective well-being in different directions (Atkinson, 1957; Elliot and Church, 1997; Elliot and McGregor, 2001; Feng and Yang, 2024). In the context of medical education, students with a higher motive to achieve success may be more likely to appraise difficult coursework, clinical training, and evaluation tasks as competence-building experiences that contribute to professional meaning, rather than merely as sources of pressure (Elliot and Church, 1997; Li et al., 2021; Luo et al., 2024). In contrast, students with a higher motive to avoid failure may be more likely to experience demanding coursework, clinical training, and evaluation tasks as stressors, making perceived stress and related negative emotional responses more salient (Lazarus and Folkman, 1984; Wang et al., 2021).

Although the direct paths from both achievement motivation dimensions to subjective well-being were statistically significant, both associations were weak (*β* = 0.060 for the motive to achieve success and *β* = −0.063 for the motive to avoid failure). Interpreting these associations therefore requires attention to the psychological resources included in the model. Existing studies have supported several partial paths linking achievement-related motivation, self-efficacy, resilience, academic outcomes, and psychological health (Hayat et al., 2021; Li et al., 2022; Qin et al., 2023). Within this conceptual framework, achievement motivation represents a general motivational orientation, whereas efficacy beliefs and resilience are psychological resources associated with well-being. The three specific statistical indirect paths were through general self-efficacy alone, psychological resilience alone, and general self-efficacy followed by psychological resilience. The practical significance of these small associations should be considered in relation to the complexity of well-being and the educational context (Prentice and Miller, 1992; Funder and Ozer, 2019).

### 4.2 Statistical indirect associations through general self-efficacy and psychological resilience separately

The fitted model identified statistical indirect associations between both dimensions of achievement motivation and subjective well-being through general self-efficacy. This finding is partly consistent with previous evidence showing that achievement motivation is associated with self-efficacy and adaptive academic outcomes among university students (Li et al., 2022). Specifically, the motive to achieve success was positively related to general self-efficacy, whereas the motive to avoid failure was negatively related to general self-efficacy. Higher general self-efficacy was also associated with higher subjective well-being. This pattern is also compatible with achievement motivation theory and recent evidence showing that approach-oriented and avoidance-oriented achievement motivation may be linked to subjective well-being in different directions (Elliot, 1999; Feng and Yang, 2024).

According to social cognitive theory, individuals’ efficacy beliefs play an important role in motivational functioning (Bandura, 1997). In medical education, students with a relatively strong motive to succeed may be more inclined to approach heavy coursework and clinical practice as opportunities for skill acquisition and personal progress, which may strengthen their sense of competence and perceived control (Pajares, 1996; Elliot and Church, 1997; Elliot, 1999; Chemers et al., 2001; Artino, 2012). By contrast, students who are highly sensitive to failure may be more prone to concerns about negative evaluation or possible failure, making it more difficult for them to believe that they can meet the demanding requirements of medical training (Elliot and Church, 1997; Elliot, 1999). When facing academic difficulties, students with higher self-efficacy are generally more likely to maintain effort and adaptive engagement, and existing studies have also shown that higher general self-efficacy is associated with better well-being and more adaptive coping, whereas lower self-efficacy is often related to poorer adaptation and more negative experiences (Chemers et al., 2001; Luszczynska et al., 2005; Caprara et al., 2006; Artino, 2012; Cattelino et al., 2023). General self-efficacy may therefore be interpreted as a cognitive correlate linking motivational tendencies with experiences of competence, control, and well-being (Caprara et al., 2006; Cattelino et al., 2023).

The model also identified statistical indirect associations between achievement motivation and subjective well-being through psychological resilience. This finding is consistent with medical-education research showing that resilience is closely associated with medical students’ psychological well-being and academic burnout (Neufeld and Malin, 2019; Yu and Chae, 2020; Liu and Cao, 2022). Students with a stronger motive to achieve success may show a greater tendency to maintain or mobilize psychological resources when facing stress, whereas students with a stronger motive to avoid failure may be more vulnerable to perceived resource depletion and weaker recovery capacity. For both motivational dimensions, the absolute indirect estimate through psychological resilience alone was numerically larger than that through general self-efficacy alone.

From the perspective of conservation of resources theory, psychological resilience can be understood as a personal resource that may help individuals cope with long-term stress and preserve or restore psychological functioning (Hobfoll, 1989; Hobfoll et al., 2018). Under the real conditions of medical education, higher psychological resilience may help medical students maintain a relatively stable adaptive state under intensive coursework, repeated examinations, and clinical uncertainty because resilience has been linked to better psychological well-being, higher life satisfaction, and lower academic burnout in this population (Neufeld and Malin, 2019; Yu and Chae, 2020; Liu and Cao, 2022; Wang et al., 2022).

### 4.3 The modeled sequential indirect path through general self-efficacy and psychological resilience

The results were consistent with H4 as a modeled sequential indirect association. Existing studies on psychological adaptation suggest that general self-efficacy and psychological resilience may be linked within a common adaptation pathway. In the present model, their joint contribution was represented by a theoretically specified sequential indirect association (Schwarzer and Warner, 2013; Qin et al., 2023).

A theoretical interpretation of the specified pathway links motivational tendency, efficacy beliefs, resilience, and emotional experience. General self-efficacy is closer to students’ current cognitive appraisal of whether they can meet learning and training requirements (Artino, 2012; Hayat et al., 2021), whereas psychological resilience reflects the capacity to mobilize resources, adapt to adversity, and recover from setbacks (Masten, 2001; Fletcher and Sarkar, 2013; Schwarzer and Warner, 2013). In the specified positive pathway, success motivation was positively associated with self-efficacy, self-efficacy was positively associated with resilience, and resilience was positively associated with well-being (Bandura, 1997; Hayat et al., 2021; Qin et al., 2023). The negative pathway began with the inverse association between failure-avoidance motivation and self-efficacy, followed by the same positive self-efficacy–resilience and resilience–well-being associations (Elliot, 1999; Hayat et al., 2021).

In medical education, this pattern is compatible with students who endorse success-oriented goals also reporting confidence in mastering knowledge and skills and greater perceived resilience (Hayat et al., 2021; Qin et al., 2023). Conversely, failure-avoidance may accompany concerns about negative outcomes and lower levels of confidence and perceived resilience (Zeng et al., 2016; Yu and Chae, 2020). Existing studies provide partial support for this framework by examining resilience as a mediator of associations between self-efficacy and mental health (Qin et al., 2023), and self-efficacy and resilience have been examined as sequential mediators in student adaptation-related outcomes (Zhang et al., 2024).

### 4.4 Practical implications

The findings suggest that motivational tendencies, efficacy beliefs, and resilience can be considered together when developing support for medical students’ subjective well-being.

First, considering the different associations between the two achievement motivation dimensions and well-being, medical education may benefit from helping students reduce an exclusive focus on “not failing” and develop more mastery- and success-oriented goals. Difficult tasks in medical training can be framed, where appropriate, as opportunities for learning and personal development rather than merely as threats of evaluation or failure (Dweck, 1986; Ames, 1992; Lazowski and Hulleman, 2016).

Second, self-efficacy is a relevant cognitive resource linking motivation with psychological adaptation. From the perspective of the basic sources of self-efficacy (Bandura, 1997), educators may support medical students’ competence beliefs by arranging learning content in progressive stages, providing clear feedback based on direct observation, and strengthening positive peer or teacher role modeling (Artino, 2012; Ramani and Krackov, 2012; Klassen and Klassen, 2018; Burgess et al., 2020). These strategies target learning experiences and perceived capability.

Third, the observed indirect association through psychological resilience suggests that resilience warrants attention as a potential personal resource. Existing systematic reviews and meta-analyses indicate that mindfulness-based interventions for medical students generally have beneficial, often small-to-moderate, effects on stress and some burnout-related outcomes (Sperling et al., 2023, 2026; Kaisti et al., 2024; Wang et al., 2024). Therefore, medical schools may consider incorporating resilience support programs into student support systems, while continuously evaluating their local implementation effects. For these programs to be useful, medical schools, clinical teachers, and mental health services should coordinate resources and avoid treating motivation, self-efficacy, and resilience as isolated intervention targets (Klein and McCarthy, 2022; Mohmand et al., 2022).

### 4.5 Research significance and limitations

Theoretically, this study integrates the two dimensions of achievement motivation within a common model of subjective well-being among medical students. The findings highlight their contrasting associations with well-being and the statistical indirect pathways involving general self-efficacy and psychological resilience. This framework connects motivational tendencies with cognitive beliefs and psychological resources. Practically, the findings may inform student support programs that address motivational tendencies and incorporate self-efficacy and resilience into well-being promotion.

This study has several limitations. First, the cross-sectional design does not allow causal conclusions or establish the temporal ordering of the observed associations. Because achievement motivation, general self-efficacy, psychological resilience, and subjective well-being were assessed at the same time, the findings describe their relationships at a single point rather than changes over time. Future longitudinal studies with at least three waves could examine the stability and direction of these associations and further evaluate the proposed sequence involving self-efficacy and resilience.

Second, participants were recruited from a single medical college, which may limit the generalizability of the findings. The educational environment, training arrangements, and student characteristics of this institution may differ from those of medical colleges in other regions. Research involving multiple institutions and more diverse student populations would help determine whether similar patterns are observed across different educational and cultural settings.

Third, all variables were measured using self-report questionnaires. Responses may be influenced by participants’ interpretation of the items, response tendencies, and social desirability. Assessing all constructs through the same method may also introduce common-method variance into the observed associations. Future studies could complement self-reported questionnaires with interviews, behavioral observations, or other appropriate sources of information to provide a more comprehensive assessment of medical students’ motivation, psychological resources, and well-being.

## 5 Conclusion

This study examined the associations between two-dimensional achievement motivation and medical students’ subjective well-being. Success motivation was positively associated with well-being, whereas failure-avoidance motivation was negatively associated with it. Both direct associations were weak. General self-efficacy and psychological resilience were involved in statistical indirect associations separately and in the specified sequence of self-efficacy followed by resilience. These findings support considering motivation, efficacy beliefs, and resilience together when studying medical students’ well-being.

## Data Availability

The datasets generated and/or analyzed during the current study are not publicly available due to privacy and ethical restrictions involving participants’ personal information. Reasonable requests to access the anonymized datasets may be directed to the corresponding author, Chuan Gao, at gaochuan516@163.com.
