# Supplementary material for: Associations between achievement motivation and subjective well-being among medical students: the chain mediating roles of general self-efficacy and psychological resilience
Source: Front Psychol. 2026 Oct 9;17:1931480. doi: 10.3389/fpsyg.2026.1931480 (PMC13690698; doi:10.3389/fpsyg.2026.1931480)
Supplement: Supplementary file 1 [file Data_Sheet_1.pdf]

# Supplementary Tables

Abbreviations. AS, motive to achieve success; AF, motive to avoid failure; GSE, general self-efficacy; PR, psychological resilience; SWB, subjective well-being. CI, confidence interval.

**Table S1** Descriptive statistics and Pearson correlations of composite scores

| Variable | Observed range | Skewness | Excess kurtosis | AS | AF | GSE | PR | SWB |
|---|---|---|---|---|---|---|---|---|
| AS | 1.000–4.000 | −0.038 | −0.401 | 1.000 | — | — | — | — |
| AF | 1.000–3.933 | 0.263 | −0.336 | −0.138<br>[−0.176, −0.098] | 1.000 | — | — | — |
| GSE | 1.000–4.000 | −0.012 | −0.412 | 0.480<br>[0.449, 0.510] | −0.155<br>[−0.194, −0.116] | 1.000 | — | — |
| PR | 1.000–4.000 | −0.180 | −0.429 | 0.399<br>[0.365, 0.432] | −0.134<br>[−0.173, −0.095] | 0.435<br>[0.403, 0.467] | 1.000 | — |
| SWB | 1.000–6.000 | −0.340 | −0.329 | 0.350<br>[0.314, 0.384] | −0.161<br>[−0.200, −0.122] | 0.414<br>[0.381, 0.447] | 0.568<br>[0.540, 0.594] | 1.000 |

Note. N = 2,422, with no missing composite scores. Observed ranges and distribution statistics refer to composite mean scores. Off-diagonal entries are Pearson correlations with 95% confidence intervals calculated using Fisher's z transformation; all correlations had $p < 0.001$.

**Table S2** Reliability, convergent validity, and discriminant validity

| Factor | Items | α | CR | AVE | Loading range | AS | AF | GSE | PR | SWB |
|---|---|---|---|---|---|---|---|---|---|---|
| AS | 15 | 0.935 | 0.935 | 0.491 | 0.677–0.721 | **0.701** | −0.147 | 0.524 | 0.431 | 0.371 |
| AF | 15 | 0.939 | 0.939 | 0.507 | 0.683–0.759 | −0.147 | **0.712** | −0.169 | −0.143 | −0.171 |
| GSE | 10 | 0.905 | 0.905 | 0.488 | 0.672–0.727 | 0.524 | −0.169 | **0.698** | 0.481 | 0.448 |
| PR | 10 | 0.912 | 0.912 | 0.509 | 0.689–0.736 | 0.431 | −0.143 | 0.481 | **0.714** | 0.610 |
| SWB | 20 | 0.951 | 0.951 | 0.491 | 0.675–0.724 | 0.371 | −0.171 | 0.448 | 0.610 | **0.701** |

Note. α, Cronbach's alpha; CR, composite reliability; AVE, average variance extracted. Loading ranges refer to standardized factor loadings. In the five-factor matrix, bold diagonal entries are the square roots of AVE, and off-diagonal entries are latent-factor correlations from the confirmatory factor analysis.

**Table S3** Item-level estimates of the measurement model

| Factor | Item | Loading B | SE(B) | Loading λ | Residual θ |
|---|---|---|---|---|---|
| AS | a1 | 1.000 | — | 0.694 | 0.518 |
| AS | a2 | 1.006 | 0.030 | 0.709 | 0.498 |
| AS | a3 | 1.005 | 0.032 | 0.681 | 0.537 |
| AS | a4 | 0.998 | 0.031 | 0.700 | 0.511 |
| AS | a5 | 0.974 | 0.031 | 0.679 | 0.539 |
| AS | a6 | 1.043 | 0.032 | 0.706 | 0.501 |
| AS | a7 | 0.979 | 0.031 | 0.677 | 0.542 |
| AS | a8 | 1.033 | 0.031 | 0.705 | 0.503 |
| AS | a9 | 1.010 | 0.031 | 0.706 | 0.501 |
| AS | a10 | 1.036 | 0.031 | 0.715 | 0.489 |
| AS | a11 | 1.011 | 0.032 | 0.687 | 0.528 |
| AS | a12 | 1.038 | 0.031 | 0.713 | 0.492 |
| AS | a13 | 1.046 | 0.031 | 0.721 | 0.480 |
| AS | a14 | 1.031 | 0.031 | 0.703 | 0.506 |
| AS | a15 | 1.003 | 0.030 | 0.717 | 0.486 |
| AF | a16 | 1.000 | — | 0.716 | 0.487 |
| AF | a17 | 0.956 | 0.028 | 0.706 | 0.502 |
| AF | a18 | 0.968 | 0.029 | 0.705 | 0.503 |
| AF | a19 | 1.043 | 0.029 | 0.759 | 0.424 |
| AF | a20 | 0.978 | 0.029 | 0.711 | 0.495 |
| AF | a21 | 0.952 | 0.027 | 0.727 | 0.471 |
| AF | a22 | 0.973 | 0.028 | 0.711 | 0.495 |
| AF | a23 | 0.951 | 0.028 | 0.700 | 0.510 |
| AF | a24 | 0.990 | 0.028 | 0.727 | 0.471 |
| AF | a25 | 0.964 | 0.029 | 0.692 | 0.521 |
| AF | a26 | 0.954 | 0.028 | 0.696 | 0.515 |
| AF | a27 | 0.923 | 0.028 | 0.683 | 0.533 |
| AF | a28 | 0.992 | 0.028 | 0.727 | 0.472 |
| AF | a29 | 0.948 | 0.028 | 0.715 | 0.488 |
| AF | a30 | 0.939 | 0.028 | 0.707 | 0.500 |
| GSE | c1 | 1.000 | — | 0.715 | 0.488 |
| GSE | c2 | 0.978 | 0.031 | 0.678 | 0.540 |
| GSE | c3 | 0.974 | 0.029 | 0.708 | 0.498 |
| GSE | c4 | 1.034 | 0.030 | 0.722 | 0.479 |
| GSE | c5 | 1.024 | 0.031 | 0.701 | 0.509 |
| GSE | c6 | 1.050 | 0.031 | 0.727 | 0.472 |
| GSE | c7 | 0.976 | 0.030 | 0.694 | 0.518 |
| GSE | c8 | 0.973 | 0.030 | 0.688 | 0.527 |
| GSE | c9 | 0.970 | 0.030 | 0.677 | 0.541 |
| GSE | c10 | 0.959 | 0.030 | 0.672 | 0.549 |
| PR | d1 | 1.000 | — | 0.721 | 0.480 |
| PR | d2 | 0.957 | 0.029 | 0.689 | 0.525 |
| PR | d3 | 0.973 | 0.029 | 0.696 | 0.515 |
| PR | d4 | 1.025 | 0.030 | 0.726 | 0.473 |

| Factor | Item | Loading B | SE(B) | Loading λ | Residual θ |
|---|---|---|---|---|---|
| PR | d5 | 1.013 | 0.029 | 0.736 | 0.458 |
| PR | d6 | 1.024 | 0.030 | 0.719 | 0.483 |
| PR | d7 | 0.991 | 0.030 | 0.703 | 0.505 |
| PR | d8 | 0.995 | 0.029 | 0.729 | 0.469 |
| PR | d9 | 0.992 | 0.029 | 0.706 | 0.501 |
| PR | d10 | 0.993 | 0.029 | 0.709 | 0.497 |
| SWB | b1 | 1.000 | — | 0.712 | 0.493 |
| SWB | b2 | 0.986 | 0.029 | 0.713 | 0.492 |
| SWB | b3 | 0.916 | 0.028 | 0.675 | 0.545 |
| SWB | b4 | 0.952 | 0.028 | 0.698 | 0.513 |
| SWB | b5 | 0.985 | 0.029 | 0.705 | 0.503 |
| SWB | b6 | 0.968 | 0.029 | 0.702 | 0.507 |
| SWB | b7 | 0.980 | 0.029 | 0.708 | 0.498 |
| SWB | b8 | 0.940 | 0.028 | 0.707 | 0.500 |
| SWB | b9 | 0.964 | 0.029 | 0.680 | 0.537 |
| SWB | b10 | 0.984 | 0.029 | 0.693 | 0.519 |
| SWB | b11 | 0.968 | 0.028 | 0.710 | 0.496 |
| SWB | b12 | 0.996 | 0.029 | 0.701 | 0.509 |
| SWB | b13 | 0.948 | 0.028 | 0.704 | 0.504 |
| SWB | b14 | 0.951 | 0.029 | 0.690 | 0.523 |
| SWB | b15 | 0.924 | 0.028 | 0.681 | 0.536 |
| SWB | b16 | 0.967 | 0.029 | 0.703 | 0.506 |
| SWB | b17 | 0.951 | 0.028 | 0.708 | 0.499 |
| SWB | b18 | 0.938 | 0.028 | 0.698 | 0.513 |
| SWB | b19 | 0.960 | 0.029 | 0.698 | 0.513 |
| SWB | b20 | 0.976 | 0.028 | 0.724 | 0.476 |

Note. B, unstandardized factor loading; SE(B), standard error of B; λ, standardized factor loading; θ, standardized residual variance. The first loading for each factor was fixed at 1.000, with its SE shown as "—". Each item loaded on one factor, with uncorrelated residuals. N = 2,422.

**Table S4** Model fit comparisons and reversed-order model estimates

| Model | χ² (df) | CFI | TLI | RMSEA [90% CI] | SRMR |
|---|---|---|---|---|---|
| Five-factor CFA | 2596.375 (2335) | 0.997 | 0.997 | 0.0068 [0.0050, 0.0083] | 0.0167 |
| Primary structural model | 2596.375 (2335) | 0.997 | 0.997 | 0.0068 [0.0050, 0.0083] | 0.0167 |
| Reversed mediator order | 2596.375 (2335) | 0.997 | 0.997 | 0.0068 [0.0050, 0.0083] | 0.0167 |
| One-factor CFA | 47796.129 (2345) | 0.477 | 0.461 | 0.0895 [0.0888, 0.0902] | 0.1344 |
| Four-factor CFA (GSE + PR) | 9326.225 (2339) | 0.920 | 0.917 | 0.0351 [0.0344, 0.0359] | 0.0429 |
| **Paths in the reversed-order model** | | | | | |
| **Path** | | | | **Standardized β** | |
| AS → PR | | | | 0.419 | |
| AF → PR | | | | −0.082 | |
| AS → GSE | | | | 0.381 | |
| AF → GSE | | | | −0.069 | |
| PR → GSE | | | | 0.306 | |
| AS → SWB | | | | 0.060 | |
| AF → SWB | | | | −0.063 | |
| PR → SWB | | | | 0.494 | |
| GSE → SWB | | | | 0.169 | |

Note. N = 2,422; all models used 70 items and maximum likelihood with a Wishart objective (MLW). CFA, confirmatory factor analysis; df, degrees of freedom; CFI, comparative fit index; TLI, Tucker–Lewis index; RMSEA, root mean square error of approximation; SRMR, standardized root mean square residual. All $\chi^2$ tests had $p < 0.001$. The four-factor CFA combined GSE and PR. The reversed-order model specified AS/AF → PR → GSE → SWB, with all corresponding direct paths retained and the AS–AF covariance freely estimated. The first three models are covariance-equivalent. Covariances were normalized by N; $\chi^2$ was calculated as N times the MLW discrepancy function. SRMR was calculated from standardized covariance residuals, including the diagonal.

**Table S5** Composite-score sensitivity analysis

| Association type | Path | Unadjusted β [95% CI] | Adjusted β [95% CI] | Δβ |
|---|---|---|---|---|
| Direct path | AS → GSE | 0.468 [0.435, 0.498] | 0.468 [0.435, 0.498] | 0.0002 |
| Direct path | AF → GSE | −0.091 [−0.128, −0.056] | −0.091 [−0.128, −0.056] | −0.0003 |
| Direct path | AS → PR | 0.242 [0.204, 0.280] | 0.242 [0.202, 0.280] | −0.0009 |
| Direct path | AF → PR | −0.053 [−0.087, −0.017] | −0.053 [−0.087, −0.017] | −0.0002 |
| Direct path | GSE → PR | 0.311 [0.273, 0.348] | 0.312 [0.274, 0.350] | 0.0015 |
| Direct path | AS → SWB | 0.078 [0.040, 0.118] | 0.079 [0.041, 0.119] | 0.0004 |
| Direct path | AF → SWB | −0.063 [−0.095, −0.030] | −0.063 [−0.095, −0.030] | −0.0001 |
| Direct path | GSE → SWB | 0.169 [0.131, 0.206] | 0.170 [0.132, 0.208] | 0.0012 |
| Direct path | PR → SWB | 0.454 [0.420, 0.488] | 0.453 [0.419, 0.487] | −0.0013 |
| Specific indirect | AS → GSE → SWB | 0.079 [0.060, 0.098] | 0.080 [0.061, 0.098] | — |
| Specific indirect | AS → PR → SWB | 0.110 [0.091, 0.131] | 0.109 [0.090, 0.130] | — |
| Specific indirect | AS → GSE → PR → SWB | 0.066 [0.056, 0.077] | 0.066 [0.056, 0.077] | — |
| Total indirect | AS → SWB | 0.255 [0.231, 0.281] | 0.255 [0.230, 0.281] | — |
| Total | AS → SWB | 0.334 [0.297, 0.370] | 0.334 [0.298, 0.371] | — |
| Specific indirect | AF → GSE → SWB | −0.015 [−0.023, −0.009] | −0.016 [−0.023, −0.009] | — |
| Specific indirect | AF → PR → SWB | −0.024 [−0.040, −0.008] | −0.024 [−0.040, −0.008] | — |
| Specific indirect | AF → GSE → PR → SWB | −0.013 [−0.019, −0.008] | −0.013 [−0.019, −0.008] | — |
| Total indirect | AF → SWB | −0.052 [−0.072, −0.033] | −0.052 [−0.072, −0.033] | — |
| Total | AF → SWB | −0.115 [−0.153, −0.078] | −0.116 [−0.153, −0.079] | — |

Note. Estimates were obtained from regression models using composite mean scores. β, standardized coefficient; Δβ, adjusted minus unadjusted β, calculated from unrounded estimates and reported for direct paths. Adjusted models included age (continuous), sex (code 1 as the reference), and entry cohort (2024 as the reference). The 95% confidence intervals were percentile bootstrap intervals based on the same 5,000 participant-level resamples for both models. N = 2,422.

**Table S6** Bootstrap confidence intervals for component percentages

| Component | % of total | 95% BC CI (%) |
|---|---|---|
| AS — Via GSE alone | 24.45 | [17.36, 32.38] |
| AS — Via PR alone | 34.07 | [27.52, 41.19] |
| AS — Via GSE → PR | 24.56 | [20.16, 30.24] |
| AS — Total indirect | 83.08 | [72.83, 94.91] |
| AS — Direct | 16.92 | [5.09, 27.17] |
| AF — Via GSE alone | 13.34 | [7.45, 23.03] |
| AF — Via PR alone | 20.50 | [5.55, 35.24] |
| AF — Via GSE → PR | 13.40 | [7.89, 22.13] |
| AF — Total indirect | 47.25 | [30.56, 68.98] |
| AF — Direct | 52.75 | [31.02, 69.44] |

Note. Percent of total = 100 × component estimate / total association for the same predictor in the primary latent-variable model. BC, bias-corrected. Confidence intervals were calculated from 5,000 participant-level bootstrap resamples, with the model re-estimated in each resample.
